# The Borderland of Insanity

**Published:** 1899-09-23

**Authors:** 


### Editor's Letter-Box.

[Our Correspondents are reminded that prolixity is a great bar to publication, and that brevity of style and conciseness of statement greatly facilitate early insertion.]

### THE BORDERLAND OF INSANITY.

"M. H. A. V." writes: There is a subject which I should much like to see discussed in your columns. It is the problem of what to do with patients who are threatened with mental derangement, or show symptoms which are suspicious, but are not pronounced enough to justify a certificate for insanity. There is no question that it is right to make it difficult to send people to asylums, and yet some terrible results have occurred through delay in doing so. I would suggest the institution of homes attached to our asylums, but bearing a different name, where patients in a doubtful condition might be sent to be under observation. If they recovered without a mental breakdown no harm is done to them socially from having been under restraint, and if they became insane they could easily be removed to the asylum to which such home was attached. There are many cases of nervous disease from overwork and other causes, not necessarily mental, but which might become so, that would be much more readily cured by removal from their own homes. Such cases are not fit subjects for hospital treatment, though at present there is no other place for them, as their friends do not wish naturally to let them bear the stigma attaching to one who had been an asylum inmate, and yet fear to run the risk of a sudden outbreak of mania. I know there are medical men who have homes for these kind of cases, but the fees are high, and no provision is made for the poorer classes and those who can only afford to pay a little. A good institution of this sort would afford a splendid training ground for the student, especially as regards his diagnostic work.

*** In the present state of the Lunacy Law it is illegal to employ restraint except under certificate, or to receive more than one certified case in "private care" except by the special permission of the Lunacy Commissioners.—Ed. *T. H.*

